# Response of amino acids, phenolic acids, organic acids, and mineral elements to fulvic acid in spinach (*Spinacia oleracea* L.) under nitrate stress

**DOI:** 10.1038/s41598-025-93974-7

**Published:** 2025-03-19

**Authors:** Kangning Han, Cheng Wang, Yanqiang Gao, Jing Zhang, Jianming Xie

**Affiliations:** College of Horticulture, Gansu Agricultural University, Yingmen Village, Anning District, Lanzhou, 730070 China

**Keywords:** Physiology, Plant sciences

## Abstract

Fulvic acid (FA) acid has many physiological activities, but the specific metabolic responses and changes in mineral element contents of spinach by FA in response to nitrate stress are unknown. Herein, we used liquid chromatography-mass spectrometry (LC–MS) and wet digestion using H_2_SO_4_-H_2_O_2_ to analyze the metabolic response and changes in the mineral element content of spinach to nitrate stress (150 mM NO_3_^−^) after FA (0.15%) foliar spray application. After 2 days of the stress treatment, FA was sprayed thrice (once every 7 days), sampled 4 days after the last spraying, and metabolites and mineral element contents were measured. FA treatment significantly increased organic acid contents (tartaric acid, malic acid, citric acid, and ascorbic acid) and amino acid contents (threonine, asparagine, valine, tyrosine, alanine, glutamate, serine, histidine, arginine, and glutamine) under nitrate stress. FA application also significantly improved mineral element contents (P, Na, Fe, and Zn) under nitrate stress. This study provides comprehensive insights into metabolite accumulation of metabolites and the improvement of nutritional quality in spinach through FA application under nitrate stress. Further research should focus on elucidating additional underlying molecular mechanisms of these metabolic responses for better utilization of this natural compound in agriculture.

Spinach (*Spinacea oleracea* L.) is an herbaceous plant in the spinach genus of the Chenopodiaceae family, also known as red root cabbage, parrot cabbage, Persian cabbage^1^. It is an essential green leafy vegetable worldwide and is accepted by consumers for its delicious flavor and rich nutrition^2^. Spinach is rich in carotenoids, vitamins (A, C, E, and K), minerals (Calcium, Ferrum, etc.), antioxidants, dietary fiber, amino acids, etc. These nutrients contribute greatly to the nutritional value of spinach. Ancient Arabs had listed it as “the king of the vegetables”^3^. In addition, these nutrients give spinach its antioxidant, anti-inflammatory, and anticancer properties^4^ and are effective in treating anemia and safeguarding intestinal health. The human body cannot synthesize eight nutritionally essential amino acids. They are obtained from foods consumed by humans, and spinach is a good source of these amino acids. Spinach is rich in phenolic acids with good nutritional function and antioxidant and other physiological activities. A previous study has revealed that chlorogenic acid exerts an evident inhibitory effect on various cancerous cells. Similarly, its antioxidant effect is powerful^5^. Organic acids are an essential class of compounds in the nutritional profile of spinach. However, the accumulation of individual acids can cause a decline in the nutritional quality of spinach. For example, oxalic acid is a toxin and anti-nutritional factor in the plant body, mostly in the form of soluble sodium and potassium salts and insoluble calcium and magnesium salts^6^. Insoluble oxalic acid in vegetables reduces the effectiveness of calcium, and calcium oxalate crystals irritate digestive tissues, causing discomfort. Therefore, regulating oxalic acid content and form in spinach to some extent is crucial. Moreover, soluble oxalic acid can combine with many mineral elements in other foods to form insoluble salts that cannot be absorbed by the intestinal tract, inhibiting the absorption of minerals such as Ca, Fe, Mg, and Cu, causing a reduction in the nutritional value of spinach. Spinach contains amino acids, phenolic acids, organic acids, and minerals that play crucial roles in its nutritional quality. Owing to population growth, the market demand for spinach has increased substantially. Thus, many growers have neglected quality to pursue high yields, causing a reduction in the nutritional value of spinach and a loss of its original flavor.

Recently, growers who pursue high spinach yields have applied large amounts of nitrogen fertilizer, causing nitrate accumulation in the soil and spinach plants and salt stress, inhibiting their growth and development, compromising product quality and safety, and affecting human health^7^. Lan et al.^8^ showed that nitrate stress causes a decrease in mineral nutrition and fruit quality in cucumbers. Nitrate application reportedly causes oxalate accumulation and mineral uptake by plants^9^. In addition, Qadir et al.^10^ have shown that high N supply reduces the content of phenolic compounds and dry matter and strongly affects phenolic acid and flavonoid contents. Therefore, nitrate stress can severely affect the nutritional quality of vegetable crops, which can affect human health. Thus, finding an effective way to minimize the damage to nutritional quality caused by nitrate stress is crucial.

There is a general trend to implement measures to improve the nutrition and flavor of vegetable crops. For example, exogenous substances such as methyl jasmonate, betaine, and fulvic acid (FA), are sprayed in appropriate concentrations to increase the nutrient content of vegetables^11–14^. FA is the best and most potent humus component^15^; as a plant growth regulator, it promotes crop growth, improves crop quality, facilitates mineral uptake and transportation, and makes plants more salt-tolerant^16^. Because of its relatively small molecular weight and large number of active functional groups, it can easily enter plant cells as a donor of plant polyphenols or as an acceptor of hydrogen, directly affecting plant redox processes^17^. Khaled et al.^18^ showed that FA, when absorbed by the crop, can promote the development of the root system, which promotes the absorption of more nutrients and water by the crop. Shi et al.^15^ showed that FA can promote the absorption and transport of essential nutrients, maintain nutrient balance, and improve the appearance and flavor of tomato fruits under Cu and Cd stress. It has also been shown that FA has a positive effect on the nutritional quality of cabbage^19^. At present, FA has been currently applied to grain crops such as rice^20^, economic crops such as peanuts^21^, and vegetable crops such as tomatoes^22^. However, reports on the application of FA to improve the nutritional quality of vegetable crops under nitrate stress are lacking.

In agricultural production, the response and adaptation mechanisms of different crops to nitrate stress are specific, and relatively few studies have been conducted on spinach, an essential leafy vegetable. In addition, spinach is sensitive to nitrate and is economically essential, and FA has potential advantages but needs to be further investigated for its role under nitrate stress in spinach. Therefore, we investigated the changes in amino acid, phenolic acid, organic acid, and mineral element contents after FA application in spinach under nitrate stress, broadening the understanding of its effects on spinach nutrient content, exploring a new approach to mitigate the damage caused by nitrate stress, and providing theoretical and technological justifications for improving the high-quality cultivation of spinach. Similarly, we used correlation, principal component, and cluster analyses to conduct an in-depth scientific analysis and study the experimental results to provide a basis for consumer and grower preference choices regarding spinach nutritional quality and to improve the use of FA in nitrate-stressed spinach.

## Results

### Effect of fulvic acid on spinach biomass under nitrate stress

**Fig. 1:**
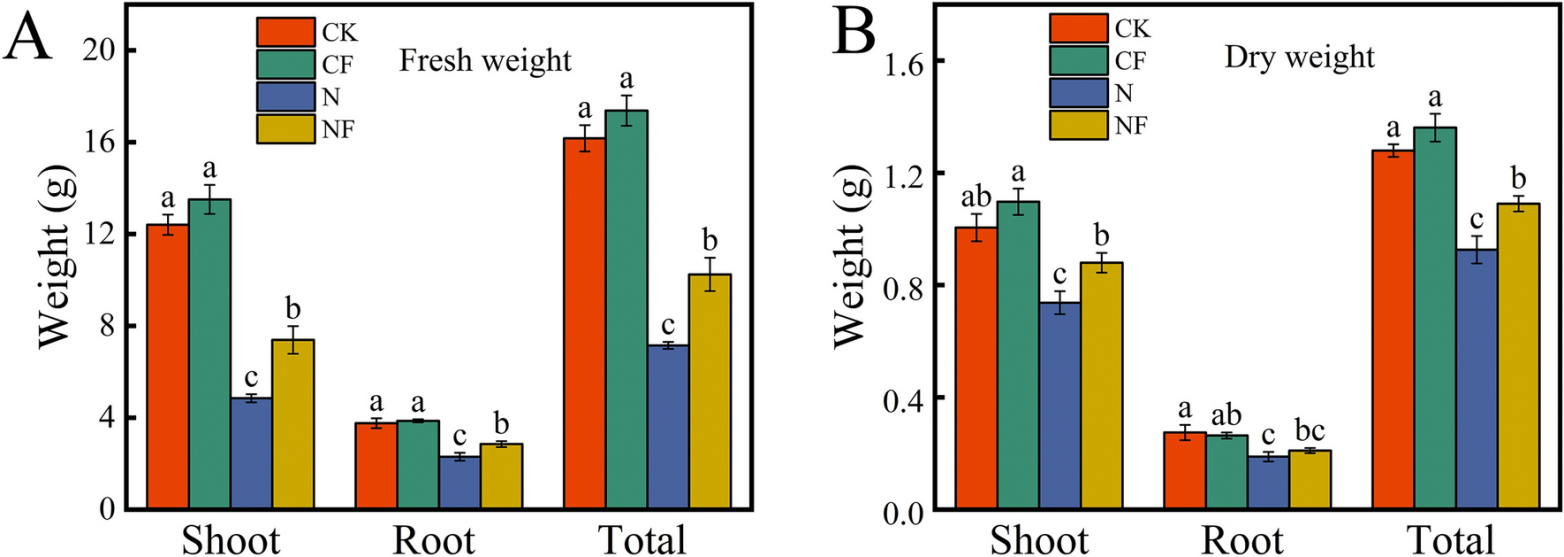
Effect of FA on spinach biomass under nitrate stress. Data are expressed as an average (n = 3). Mean values of different letters indicate significant differences using Duncan’s test (*p* < 0.05). Control (CK), 0.15% FA (CF), 150 mM NO_3_^−^ (N), 0.15% FA + 150 mM NO_3_^−^ (NF). (**A**) Fresh weight. (**B**) Dry weight.

Nitrate stress significantly reduced the fresh (Fig. 1A) and dry (Fig. 1B) weights of spinach. The total fresh and dry weights of spinach were reduced by 55.78% and 27.62%, respectively, compared with that of the control. FA application significantly alleviated the reduction in spinach biomass owing to stress. FA application increased the total fresh and dry weights of spinach by 43.26% and 17.72%, respectively, compared with those obtained during nitrate stress. This indicates that FA promotes the normal growth of spinach under nitrate stress.

### Effect of fulvic acid amino acid content in spinach under nitrate stress

FA affected the amino acid content of spinach leaves under nitrate stress (Table 1). Compared with the normal control (CK), the 150 mM NO_3_^−^ (N) treatment significantly reduced the total amino acid content by 31.34%. Phenylalanine (17.65%), threonine (21.29%), asparagine (31.99%), leucine (31.28%), isoleucine (8.62%), tryptophan (10.76%), valine (50.97%), tyrosine (45.96%), cysteine (12.27%), alanine (38.65%), glutamate (39.63%), glycine (3.00%), serine (30.34%), aspartate (38.95%), histidine (22.10%), and glutamine (8.86%) contents were significantly reduced; proline and arginine contents increased significantly by 13.99% and 32.87%. Compared with the N treatment, the 0.15% FA + 150 mM NO_3_^−^ (NF) treatment increased the total amino acid content by 11.80%. Threonine (9.44%), asparagine (27.85%), valine (12.35%), tyrosine (12.73%), alanine (6.79%), glutamate (16.84%), serine (9.57%), histidine (18.31%), arginine (51.33%), and glutamine (4.54%) contents were significantly increased; proline was decreased by 7.01%. Compared with the CK, the 0.15% FA (CF) treatment increased the total amino acid content by 5.30%. Asparagine (8.61%), valine (9.71%), proline (20.59%), tyrosine (20.93%), cysteine (13.36%), serine (23.37%), and aspartate (13.35%) contents were significantly increased, whereas tryptophan content was significantly decreased by 4.21%.

**Table 1:** Effect of FA on amino acid content (mg g^−1^ DW) in spinach under nitrate stress. Note: Data represent mean ± standard error (n = 3), and different letters in the same column indicate significant differences (*p* < 0.05). Control (CK), 0.15% FA (CF), 150 mM NO_3_^−^ (N), 0.15% FA + 150 mM NO_3_^−^ (NF).

Amino acid type	Treatments			
	CK	CF	N	NF
Phenylalanine	0.0595 ± 0.0006a	0.0591 ± 0.0020a	0.0490 ± 0.0008b	0.0495 ± 0.0007b
Threonine	0.0498 ± 0.0011a	0.0491 ± 0.0014a	0.0392 ± 0.0002c	0.0429 ± 0.0005b
Asparagine	0.0697 ± 0.0002b	0.0757 ± 0.0010a	0.0474 ± 0.0010d	0.0606 ± 0.0009c
Leucine	0.0585 ± 0.0013a	0.0557 ± 0.0051a	0.0402 ± 0.0024b	0.0403 ± 0.0010b
Isoleucine	0.0905 ± 0.0012a	0.0892 ± 0.0027a	0.0827 ± 0.0009b	0.0816 ± 0.0007b
Tryptophan	0.1283 ± 0.0023a	0.1229 ± 0.0004b	0.1145 ± 0.0009c	0.1142 ± 0.0003c
Methionine	0.0746 ± 0.0002a	0.0743 ± 0.0000a	0.0744 ± 0.0002a	0.0742 ± 0.0001a
Valine	0.1750 ± 0.0036b	0.1920 ± 0.0038a	0.0858 ± 0.0017d	0.0964 ± 0.0016c
Proline	0.1365 ± 0.0031c	0.1646 ± 0.0031a	0.1556 ± 0.0037ab	0.1447 ± 0.0046bc
Tyrosine	0.6412 ± 0.0060b	0.7754 ± 0.0100a	0.3465 ± 0.0013d	0.3906 ± 0.0065c
Cysteine	0.2575 ± 0.0043b	0.2919 ± 0.0021a	0.2259 ± 0.0014c	0.2265 ± 0.0028c
Alanine	0.3480 ± 0.0057a	0.3388 ± 0.0011a	0.2135 ± 0.0033c	0.2280 ± 0.0055b
Glutamate	2.8215 ± 0.0595a	2.6842 ± 0.0816a	1.7033 ± 0.0351c	1.9902 ± 0.0404b
Glycine	0.1735 ± 0.0004a	0.1738 ± 0.0010a	0.1683 ± 0.0013b	0.1680 ± 0.0005b
Serine	0.3748 ± 0.0034b	0.4624 ± 0.0076a	0.2611 ± 0.0079d	0.2861 ± 0.0028c
Aspartate	2.1805 ± 0.0091b	2.4716 ± 0.0437a	1.3313 ± 0.0518c	1.4366 ± 0.0061c
Histidine	0.1353 ± 0.0025a	0.1365 ± 0.0017a	0.1054 ± 0.0024c	0.1247 ± 0.0017b
Arginine	0.2519 ± 0.0056c	0.2627 ± 0.0105c	0.3347 ± 0.0110b	0.5065 ± 0.0160a
Cystine	0.2826 ± 0.0006a	0.2835 ± 0.0007a	0.2840 ± 0.0010a	0.2817 ± 0.0002a
Glutamine	0.1884 ± 0.0028a	0.1843 ± 0.0036ab	0.1717 ± 0.0005c	0.1795 ± 0.0004b
Total amino acids	8.4976 ± 0.0755b	8.9478 ± 0.1450a	5.8345 ± 0.0963d	6.5228 ± 0.2552c

**Fig. 2:**
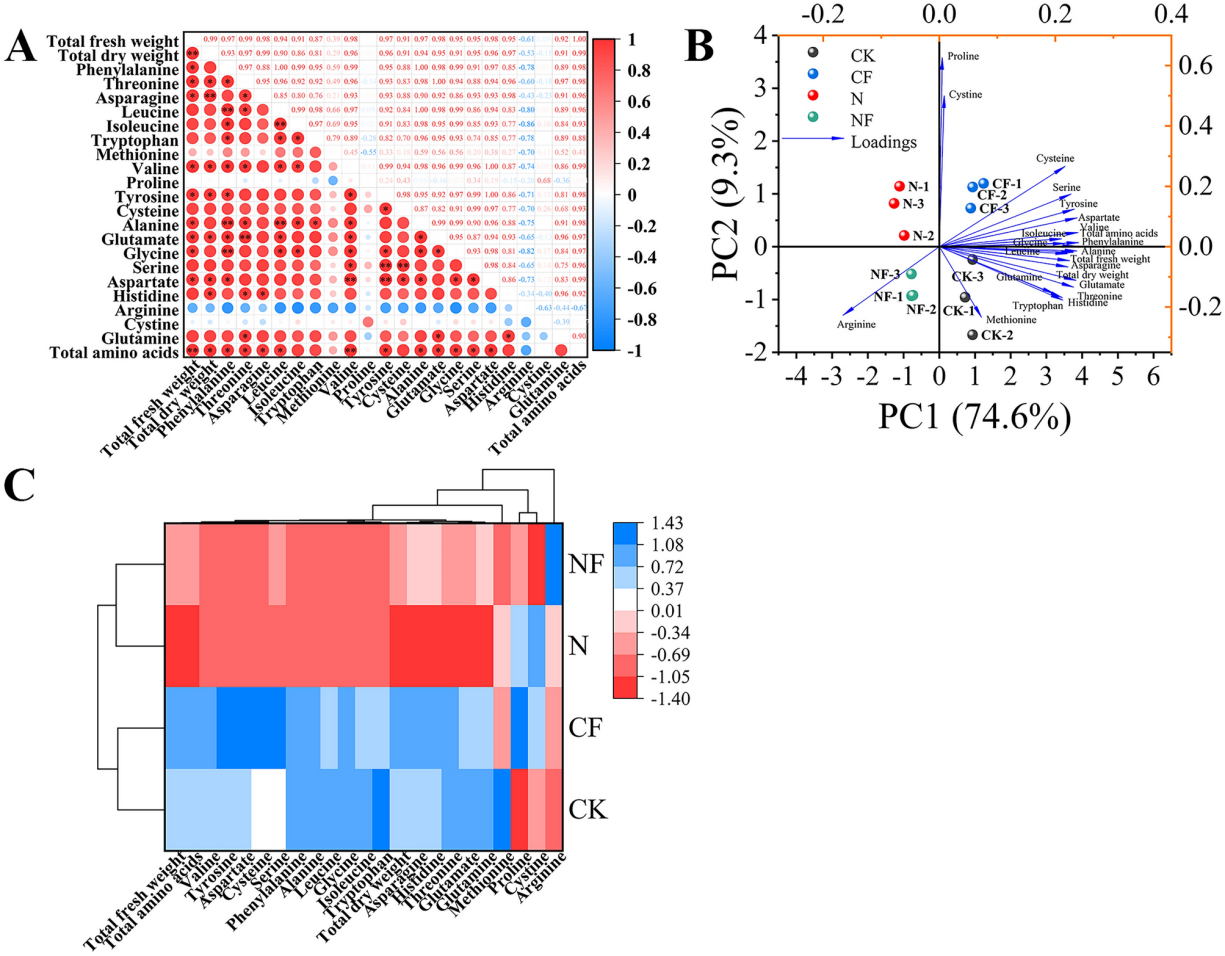
Pearson’s correlation, principal component, and cluster analyses of amino acids in spinach after FA treatment under nitrate stress. Data is expressed as an average (n = 3). * and ** showed significant correlation at *p* < 0.05 and *p* < 0.01. Control (CK), 0.15% FA (CF), 150 mM NO_3_^−^ (N), 0.15% FA + 150 mM NO_3_^−^ (NF). (**A**) Pearson’s correlation analysis. (**B**) Principal component analysis. (**C**) Cluster analysis; the scale represents different intervals of the data.

The data presented in Fig. 2A illustrate the correlation analysis of the amino acid components with each other. Highly significant positive correlations were observed between threonine and glutamate (r = 1.00), leucine and alanine (r = 1.00), and leucine and isoleucine (r = 0.99). Valine was highly significantly and positively correlated with aspartate (r = 1.00). Tyrosine was highly significantly and positively correlated with aspartate (r = 1.00) and serine (r = 0.99). Cysteine was highly significantly and positively correlated with serine (r = 0.99). Additionally, the total fresh weight was highly significantly and positively correlated with the total amino acid content (r = 1.00). Total dry weight was highly significantly and positively correlated with asparagine (r = 0.99). This indicates that amino acid accumulation and fresh weight may promote each other during spinach growth and that asparagine may be crucial for spinach dry matter accumulation.

Classification of the amino acid content of spinach under nitrate stress was based on principal component analysis (Fig. 2B). Four treatments with 21 amino acid parameters and two biomass parameters formed the corresponding taxa. Two principal components were selected from the 23 indices, with the first and second components accounting for 74.6% and 9.3% (83.9%) of the total variance, respectively. In addition, the loading plot shows that total fresh weight, total dry weight, valine, phenylalanine, alanine, asparagine, and leucine had strong first PC loadings. This indicates that the increase in fresh and dry weights varies synergistically with the accumulation of these amino acids during spinach growth. Proline and cysteine had strong secondary principal component loadings. Arginine loaded negatively on PC1 and PC2. Methionine was negatively loaded onto PC2. The CK, CF, N, and NF treatments produced a clear separation on PC1. The classification model based on cluster analysis was used to divide the four treatments into two primary categories: the CK and CF treatments and the N and NF treatments (Fig. 2C).

### Effect of fulvic acid on phenolic acid content in spinach under nitrate stress

**Fig. 3:**
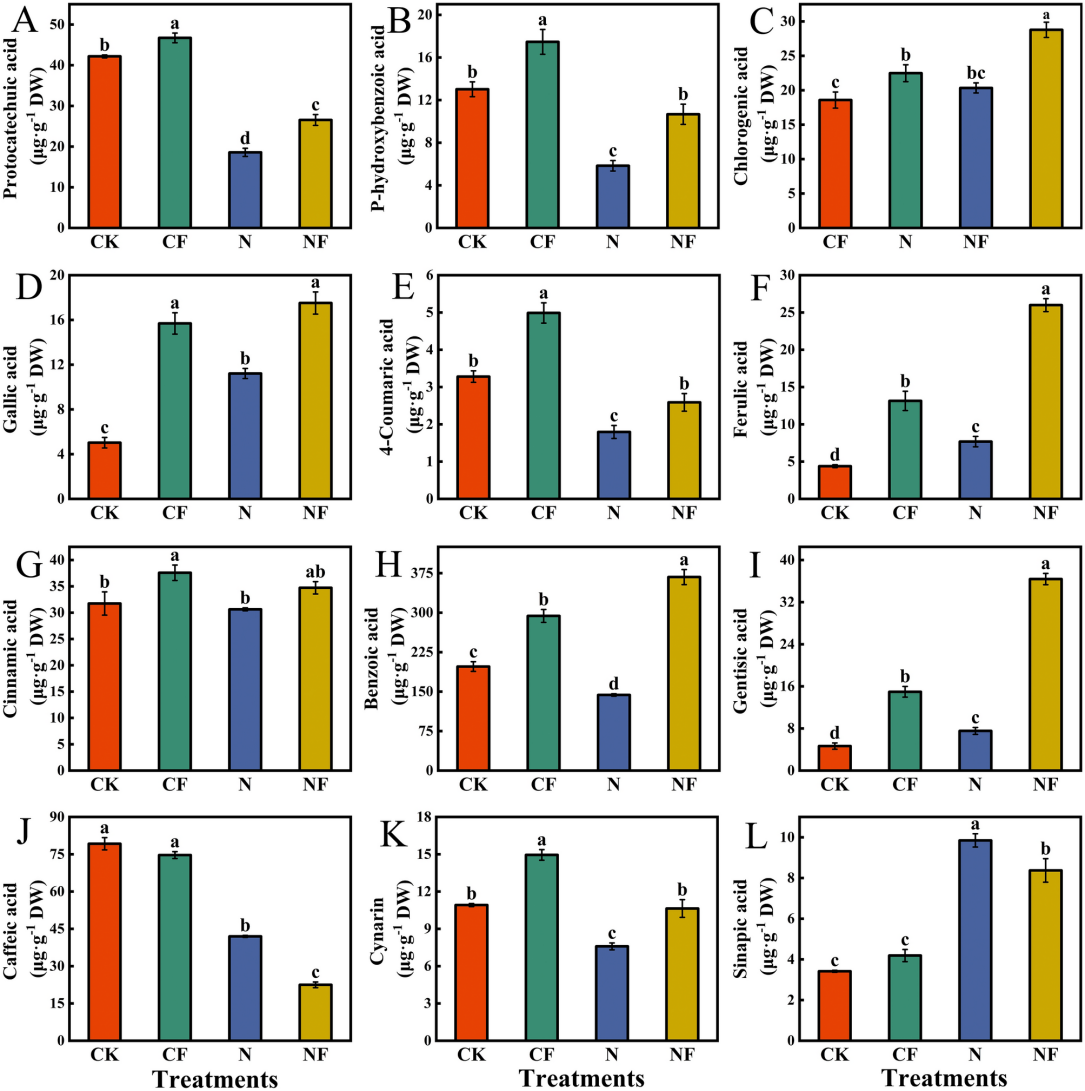
Effect of FA on phenolic acid content in spinach under nitrate stress. Data are expressed as an average (n = 3). Mean values of different letters indicate significant differences using Duncan’s test (*p* < 0.05). Control (CK), 0.15% FA (CF), 150 mM NO_3_^−^ (N), 0.15% FA + 150 mM NO_3_^−^ (NF). (**A**) Protocatechuic acid. (**B**) p-hydroxybenzoic acid. (**C**) Chlorogenic acid. (**D**) Gallic acid. (**E**) 4-coumaric acid. (**F**) Ferulic acid. (**G**) Cinnamic acid. (**H**) Benzoic acid. (**I**) Gentisic acid. (**J**) Caffeic acid. (**K**) Cynarin. (**L**) Sinapic acid.

FA affected the phenolic acid content of spinach leaves under nitrate stress (Fig. 3). Compared with the CK, the N treatment significantly reduced protocatechuic acid (55.93%), p-hydroxybenzoic acid (55.15%), 4-coumaric acid (45.12%), benzoic acid (27.31%), caffeic acid (47.01%), and cynarin (30.49%) content, and gallic, ferulic, gentisic, and sinapic acid contents were significantly increased by 122.86%, 75.17%, 61.59% and 188.01%, respectively. Compared with the N treatment, the NF treatment significantly increased the protocatechuic acid (42.76%), p-hydroxybenzoic acid (82.71%), 4-coumaric acid (43.89%), benzoic acid (155.87%), and cynarin (40.18%) contents; caffeic and sinapic acid contents were significantly reduced by 46.44% and 15.03%, respectively. Compared with CK, CF treatment significantly increased protocatechuic acid (10.76%), p-hydroxybenzoic acid (34.18%), chlorogenic acid (20.99%), gallic acid (211.93%), 4-coumaric acid (42.99%), ferulic acid (199.54%), cinnamic acid (18.40%), benzoic acid (48.65%), gentisic acid (221.24%), and cynarin (36.90%) content.

**Fig. 4:**
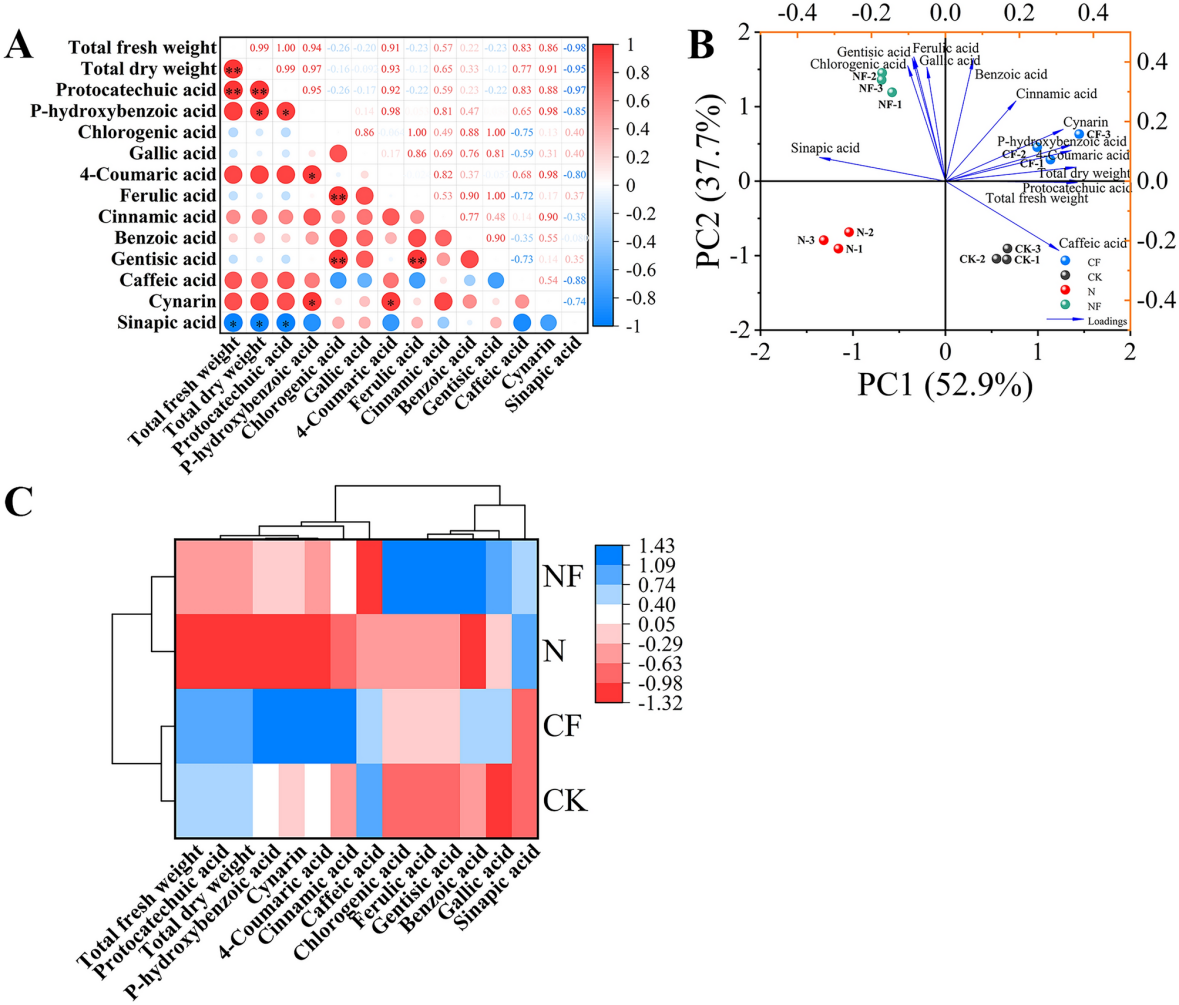
Pearson’s correlation, principal component, and cluster analyses of phenolic acids in spinach after FA treatment under nitrate stress. Data is expressed as an average (n = 3). * and ** showed significant correlation at *p* < 0.05 and* p* < 0.01. Control (CK), 0.15% FA (CF), 150 mM NO_3_^−^ (N), 0.15% FA + 150 mM NO_3_^−^ (NF). (**A**) Pearson’s correlation analysis. (**B**) Principal component analysis. (**C**) Cluster analysis; the scale represents different intervals of the data.

The data presented in Fig. 4A illustrate the correlation analysis of the phenolic acid components with each other. Protocatechuic acid showed a significant positive correlation with p-hydroxybenzoic acid (r = 0.95) and a significant negative correlation with sinapic acid (r = −0.97). p-hydroxybenzoic acid showed a significant positive correlation with cynarin (r = 0.98) and 4-coumaric acid (r = 0.98). Highly significant positive correlations were found between chlorogenic and gentisic acids (r = 1.00), chlorogenic and ferulic acids (r = 1.00), and ferulic and gentisic acids (r = 1.00). Furthermore, 4-coumaric acid showed a significant positive correlation with cynarin (r = 0.98). Additionally, total fresh and dry weights were highly significantly and positively correlated with protocatechuic acid (r = 1.00, r = 0.99). Total fresh and dry weights were significantly negatively correlated with sinapic acid content (r = −0.98, r = −0.95). This indicates that the synthesis of protocatechuic acid was promoted along with biomass accumulation during spinach growth, whereas there was an antagonistic relationship between biomass and sinapic acid accumulation, possibly because the synthesis of other components is prioritized during metabolic processes, causing a reduction in the accumulation of sinapic acid.

The principal component analysis of the influence of FA on phenolic acid content in spinach under nitrate stress is shown in Fig. 4B. Four treatments with 12 phenolic acid components and two biomass parameters formed the corresponding taxonomic groups. Two principal components were selected from the 14 indicators, with the first and second components accounting for 52.9% and 37.7% (90.6%) of the total variance, respectively. In addition, the loading diagram shows that benzoic acid, gallic acid, ferulic acid, gentisic scid and chlorogenic acid had strong secondary principal component loadings. Total fresh weight, total dry weight, protocatechuic acid, 4-coumaric acid, p-hydroxybenzoic acid, and cynarin had strong first principal component loadings. Sinapic acid had a negative loading on PC1. The CK, CF, N, and NF treatments were significantly separated on PC1. The classification model based on cluster analysis was used to divide the four treatments into two primary categories: the CK and CF treatments and the N and NF treatments (Fig. 4C).

### Effect of fulvic acid on organic acid content in spinach under nitrate stress

**Fig. 5:**
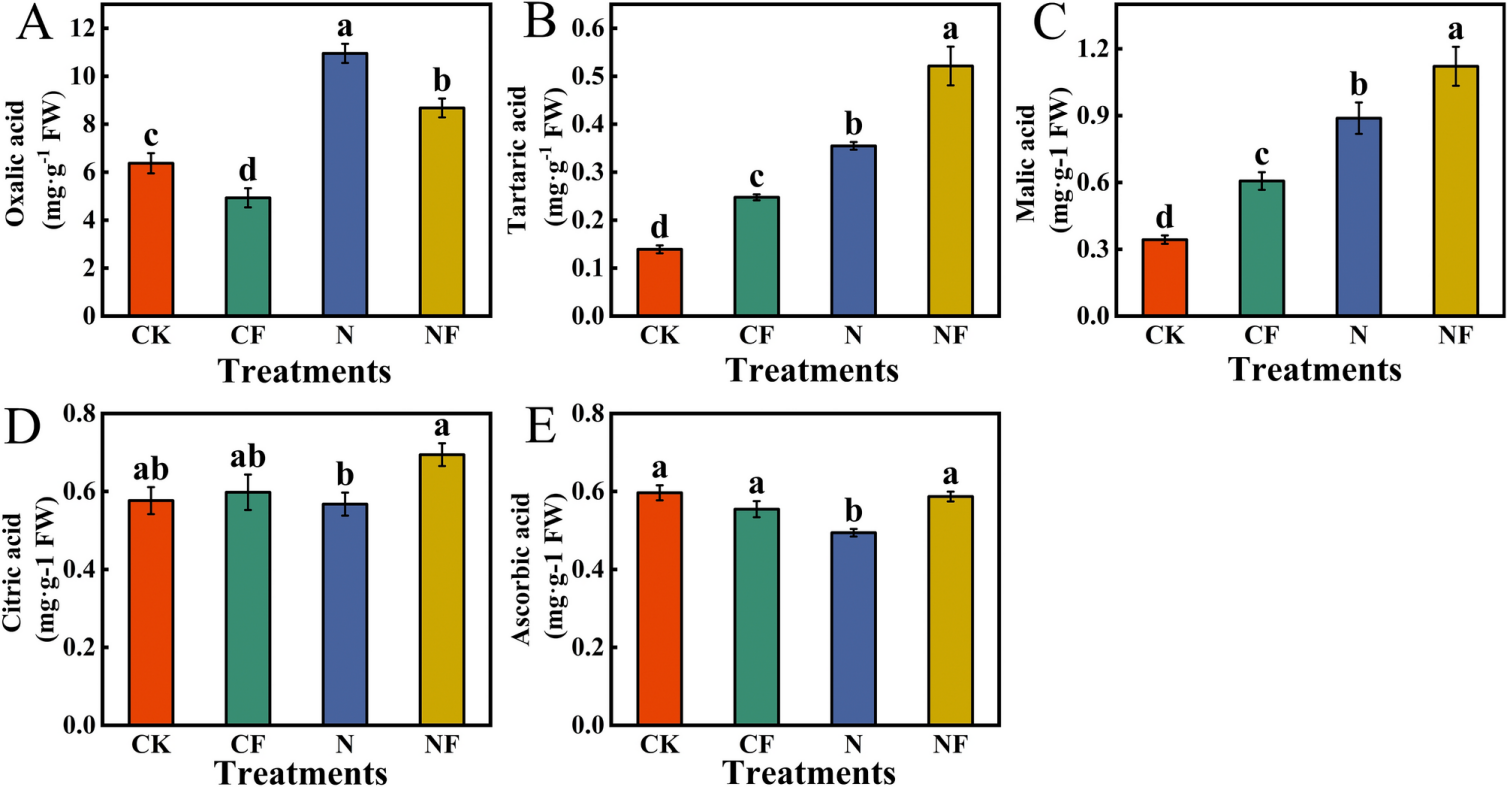
Effect of FA on organic acid content in spinach under nitrate stress. Data are expressed as an average (n = 3). Mean values of different letters indicate significant differences using Duncan’s test (*p* < 0.05). Control (CK), 0.15% FA (CF), 150 mM NO_3_^−^ (N), 0.15% FA + 150 mM NO_3_^−^ (NF). (**A**) Oxalic acid. (**B**) Tartaric acid. (**C**) Malic acid. (**D**) Citric acid. (**E**) Ascorbic acid.

FA affected the organic acid content of spinach under nitrate stress (Fig. 5). Compared with CK, N treatment significantly increased oxalic, tartaric, and malic acid contents by 72.06%, 155.40%, and 159.18%, respectively; ascorbic acid content was significantly decreased by 17.25%. Compared with the N treatment, the NF treatment significantly increased tartaric, malic, citric, and ascorbic acid contents by 46.76%, 26.21%, 22.18%, and 18.83%, respectively, and oxalic acid content was significantly reduced by 20.80%. Compared with CK, CF treatment significantly increased tartaric and malic acid contents by 78.42% and 76.97%; oxalic acid content was significantly reduced by 22.61%.

**Fig. 6:**
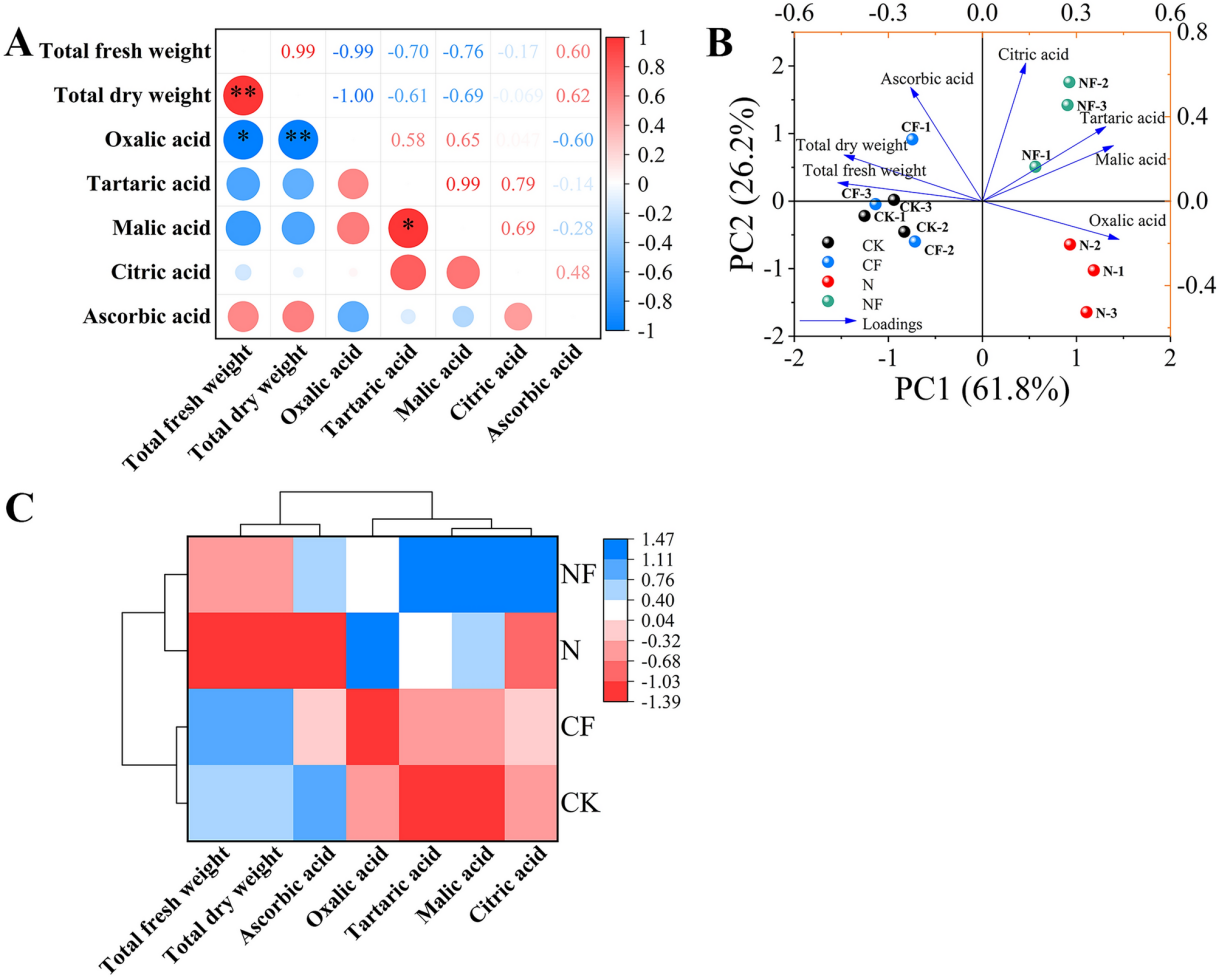
Pearson’s correlation, principal component, and cluster analyses of organic acids in spinach after FA treatment under nitrate stress. Data are expressed as average values (n = 3). The data is expressed as an average (n = 3). * showed significant correlation at *p* < 0.05. Control (CK), 0.15% FA (CF), 150 mM NO_3_^−^ (N), 0.15% FA + 150 mM NO_3_^−^ (NF). (**A**) Pearson’s correlation analysis. (**B**) Principal component analysis. (**C**) Cluster analysis; the scale represents different intervals of the data.

The data presented in Fig. 6A illustrate the correlation analysis of the organic acid components with each other. Tartaric acid significantly and positively correlated with malic acid (r = 0.99). The other organic acid components were not significantly correlated. Additionally, total fresh weight showed a significant negative correlation with oxalic acid (r = −0.99). Total dry weight was highly significantly and negatively correlated with oxalic acid (r = −1.00). This indicates that oxalic acid content and spinach biomass accumulation may be regulated by different physiological mechanisms and that these mechanisms tend to exhibit a trade-off relationship between the two under different treatment conditions.

The principal component analysis of the effect of FA on organic acid content in spinach under nitrate stress is shown in Fig. 6B. Four treatments with 5 organic acid components and two biomass parameters formed the corresponding taxonomic groups. Two principal components were selected from the7 indicators, with the first and second components accounting for 61.8% and 26.2% (88.0%) of the total variance, respectively. In addition, the loading plots showed that ascorbic acid and citric acid had strong secondary principal component loadings. Tartaric acid and malic acid had strong first principal component loadings. Oxalic acid had a negative loading on PC2. Total fresh and dry weights had positive loadings on PC2. The CF treatment did not cause a clear separation from the CK treatment. In contrast, the CK and CF treatments were clearly separated from the N and NF treatments on PC1. A classification model based on cluster analysis was used to divide the four treatments into two primary categories: the CK and CF treatments, and the N and NF treatments (Fig. 6C).

### Effect of fulvic acid on mineral element contents in spinach under nitrate stress

**Fig. 7:**
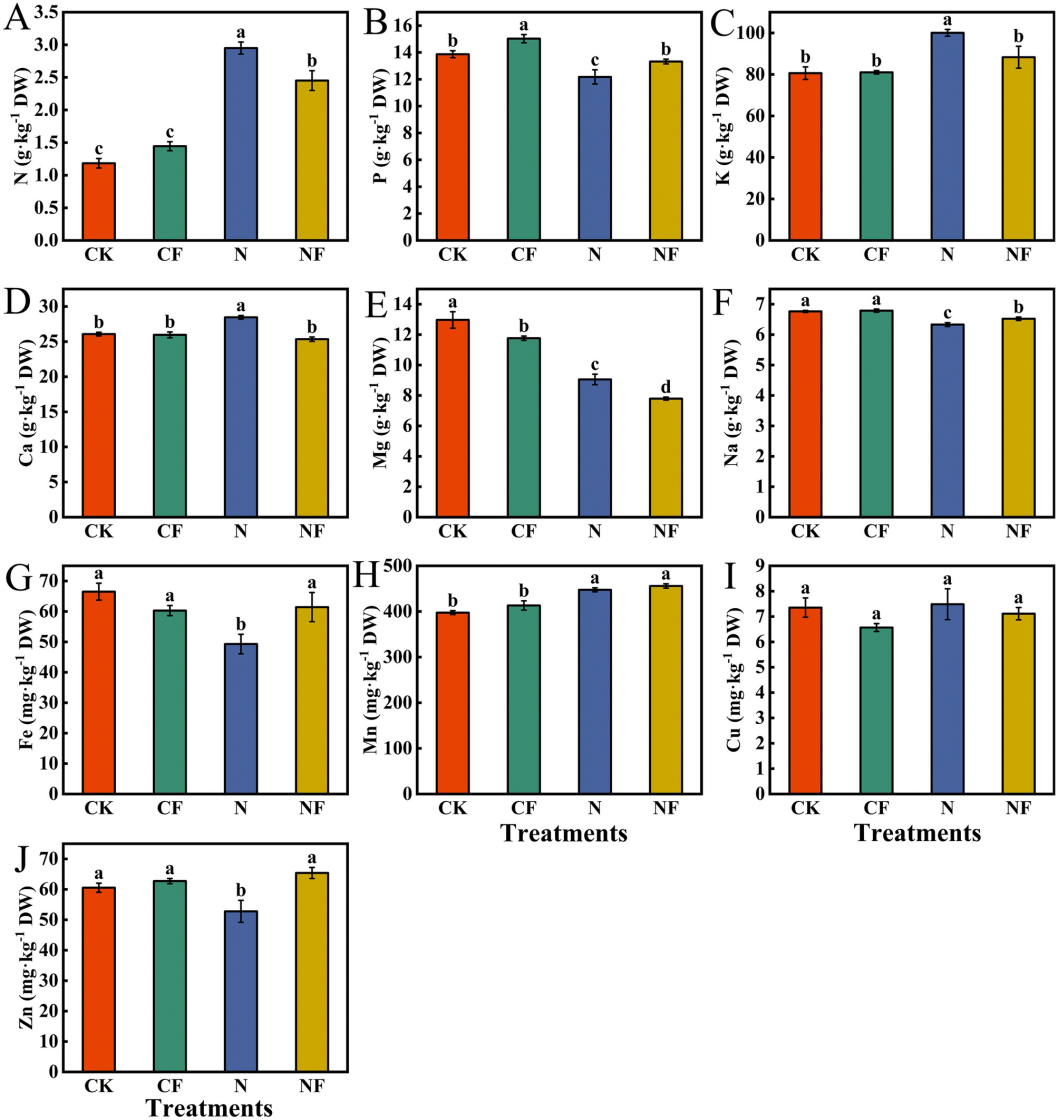
Effect of FA on the mineral element contents in spinach under nitrate stress. Data are expressed as an average (n = 3). Mean values of different letters indicate significant differences using Duncan’s test (*p* < 0.05). Control (CK), 0.15% FA (CF), 150 mM NO_3_^−^ (N), 0.15% FA + 150 mM NO_3_^−^ (NF). (**A**) Nitrogen. (**B**) Phosphorus. (**C**) Kalium. (**D**) Calcium. (**E**) Magnesium. (**F**) Natrium. (**G**) Ferrum. (**H**) Manganese. (**I**) Cuprum. (**J**) Zinc.

The effect of FA on the mineral element content of spinach under nitrate stress is shown in Fig. 7. Compared with the CK, the N treatment significantly increased nitrogen (150.00%), kalium (24.13%), calcium (9.16%), and manganese (12.58%) contents, and phosphorus (12.18%), magnesium (30.15%), natrium (6.36%), ferrum (25.89%), and zinc (12.83%) contents were significantly decreased. Compared with the N treatment, the NF treatment significantly increased phosphorus (9.36%), natrium (1.26%), ferrum (24.60%), and zinc (12.62%) contents and significantly decreased nitrogen (16.95%), kalium (11.74%), calcium (10.96%), and magnesium (14.02%) contents. Compared with CK, the CF treatment significantly increased the phosphorus content by 8.36% and significantly decreased the magnesium content by 9.33%.

**Fig. 8:**
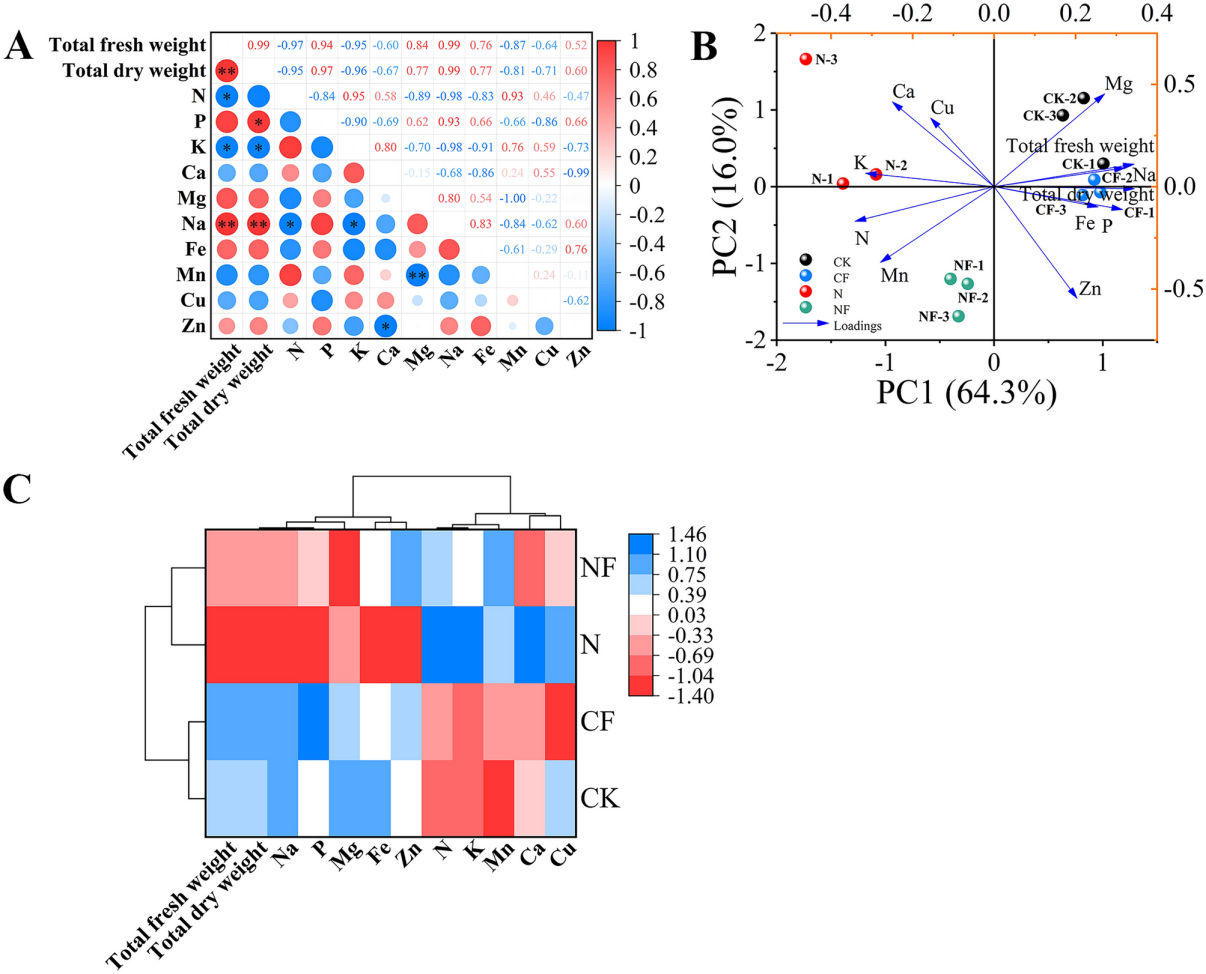
Pearson’s correlation, principal component, and cluster analyses of mineral elements in spinach after FA treatment under nitrate stress. Data is expressed as an average (n = 3). * and ** showed significant correlation at *p* < 0.05 and *p* < 0.01. Control (CK), 0.15% FA (CF), 150 mM NO_3_^−^ (N), 0.15% FA + 150 mM NO_3_^−^ (NF). (**A**) Pearson’s correlation analysis. (**B**) Principal component analysis. (**C**) Cluster analysis; the scale represents different intervals of the data.

The data presented in Fig. 8A illustrate the correlation analysis of the mineral elements with each other. Na was significantly negatively correlated with N (r = −0.98) and K (r = −0.98). Zn was significantly negatively correlated with Ca (r = −0.99). Mg was highly significantly negatively correlated with Mn (r = −1.00). Additionally, total fresh and dry weights showed a highly significant positive correlation with Na (r = 0.99, r = 0.99). Total fresh and dry weights were significantly negatively correlated with K (r = −0.95, r = −0.96). Total fresh weight was significantly negatively correlated with N (r = −0.97).

The principal component analysis of the effect of FA on the mineral element contents in spinach under nitrate stress is shown in Fig. 8B. Four treatments with 10 mineral elements and two biomass parameters formed the corresponding taxonomic groups. Two principal components were selected from the 12 indicators, with the first and second components accounting for 64.3% and 16.0% (80.3%) of the total variance, respectively. In addition, the loading plots indicated strong first principal component loadings for total fresh weight, total dry weight, Na, P, and Fe, and strong negative first principal component loadings for K and N. This indicates that Na, P, and Fe play a positive role in promoting plant growth and increasing biomass during spinach growth. There may be an antagonistic relationship between the mechanisms of N and K absorption or utilization and the mechanism of spinach biomass increase. Mg had positive loadings in PC1 and PC2. Mn showed negative loadings on PC1 and PC2. The CF treatment did not produce a significant separation from the CK treatment. In contrast, the N and NF treatments were significantly separated in PC2. A classification model based on cluster analysis was used to divide the four treatments into two primary categories: the CK and CF treatments, and the N and NF treatments (Fig. 8C).

## Discussion

Amino acids are the basic components of proteins that are required for human nutrition. The human body requires approximately 20 nutritional amino acids; of these, eight cannot be generated through the body’s metabolism and should be supplied through food proteins. Therefore, amino acids are crucial indicators of the nutritional quality of vegetables. In addition, amino acids participate in plant responses to stress via osmotic regulation^23^. It has been shown that NaCl stress reduces the amino acid content of spinach and negatively affects its nutritional quality^24^. Similar results were obtained in this study. In the present study, the application of FA application under nitrate stress increased the total amino acid content and promoted primary metabolism, which improved spinach’s nutritional value. Similarly, nitrate stress significantly increased proline and arginine content, which may be because of their roles as osmoregulators in maintaining osmotic balance under abiotic stresses, increasing plant resilience (Table 1). Arginine content also increased significantly after FA application. In our study, nitrate stress significantly reduced alanine and glutamate contents, which are associated with umami^25^, and their contents were significantly increased after FA application, suggesting that stress reduced the freshness of spinach while FA enriched its umami. This might be because FA significantly increases relevant amino acid content by promoting relevant metabolic pathways or regulating physiological processes, which enriches the fresh flavor of spinach. Li et al.^26^ showed that under cadmium stress, FA increased the amino acid content and played a central role in scavenging reactive oxygen species (ROS), reducing intracellular oxidative damage. This finding is consistent with the results of this study. Oxidative stress impairs the mitochondrial oxidative defense system, causing the disruption of the tricarboxylic acid (TCA) cycle and affecting amino acid metabolism, resulting in energy deficiency^27^. Changes in amino acid abundance affect plant adaptations to NaCl stress^28^. In this study, FA promoted tyrosine accumulation under nitrate stress, reducing oxidative damage. FA may activate metabolic pathways associated with tyrosine synthesis, prompting the conversion of more precursors to tyrosine, and the accumulated tyrosine may be directly involved in scavenging reactive oxygen radicals as an antioxidant^29^. Effective measures should be taken to mitigate the harmful effects of nitrate stress on spinach metabolism; for example, increasing the intermediate and amino acid contents of the TCA cycle can maintain the normal metabolic process under stress. In this study, FA increased threonine, asparagine, valine, tyrosine, alanine, glutamate, serine, histidine, cystine, and glutamine contents under nitrate stress. The decrease in aspartic and glutamic acid contents under nitrate stress may be owing to their rapid conversion to useful products or their binding to proteins to resist adversity^30^. Furthermore, glutamate is crucial in amino acid metabolism and is a precursor of other amino acids; changes in its content can affect the abundance of downstream amino acids. Di Martino et al.^31^ found that NaCl stress reduced the abundance of various amino acids (e.g., glycine and serine), which is consistent with our results. Moreover, studies have also shown that the accumulation of aspartic acid and arginine can reduce oxidative damage to maintain the osmotic pressure difference between cells under abiotic stress^26,32^. In this study, FA application also promoted the accumulation of the above two amino acids under nitrate stress. This suggests that FA might also improve the nutritional quality of spinach by promoting antioxidant metabolism and accelerating the accumulation of primary metabolites. This study primarily shows that FA could increase the total amino acid content of spinach under nitrate stress, promote primary metabolism, and improve the nutritional value of spinach, and revealed the role of amino acids in plant response to stress as osmotic regulating factors in maintaining osmotic balance, and in resistance to adversity. Effective measures are proposed to mitigate the harmful effects of nitrate stress on spinach metabolism, providing directions for future studies and practical applications.

Phenolic acids are vital for studying plant stress tolerance and nutrient metabolism owing to their antioxidant effects. Phenolic acid can effectively alleviate the damage caused by ROS accumulation in plants and can act as an electron transporter, promoting the transfer of electrons from the antioxidant system to ROS, and act as an electron donor in the detoxification mechanism of organelles^33^. In addition, phenolic acids, which are major plant bioactive compounds, have a wide range of biological functions and can respond to various biotic and abiotic stresses^34^. Here, nitrate stress increased and decreased the phenolic acid content to varying degrees, whereas FA application inhibited the extent of this decrease and further increased some of the phenolic acid content. Among them, gallic, ferulic, gentisic, and sinapic acid contents were increased significantly under nitrate stress. Possibly owing to nitrate stress, photosynthesis is restricted, causing excessive ROS production. To adapt to these harmful environmental conditions, plants induce the synthesis of some secondary metabolites to resist stress. Similar results have been reported in previous studies^35^. FA application caused a further increase in gallic, ferulic, and gentisic acid contents under nitrate stress. FA possibly activates key enzymes that synthesize these acids and acts as an antioxidant to mitigate the oxidative damage caused by nitrate stress, inducing the plant to increase the synthesis of these acids to resist stress. Radi et al.^36^ showed that NaCl stress could inhibit the production of binding phenolic compounds in wheat and soybean. This finding is similar to that of this study. In this study, protocatechuic acid, p-hydroxybenzoic acid, 4-coumaric acid, benzoic acid, and cynarin contents were significantly reduced under nitrate stress. Possibly because of excessive stress, the content of some phenolic acids was decreased, indicating that spinach adjusts its metabolites in response to environmental stress in a dose- and species-dependent manner^37^. FA application inhibited the reduction in their contents and promoted the accumulation of secondary metabolites under nitrate stress. Furthermore, we observed a decrease in caffeic acid content under nitrate stress, which was further reduced through FA application, possibly because of its massive depletion by spinach in response to adversity. Thus, the individual components of phenolic acids play an essential role in plant stress tolerance and nutrient metabolism owing to their antioxidant effects.

Organic acids are organic compounds containing carboxyl groups and are found in living organisms. They are acidic components of fruits and vegetables that can directly affect their taste, flavor, and nutritional quality^38^. Organic acids participate in photosynthesis, respiration, and the metabolism of vegetables and the synthesis of phenols, amino acids, esters, and aromatic substances^39^. They soften blood vessels, regulate intestinal function, promote trace element absorption, and promote the TCA cycle, which has anti-fatigue effects^40^. Some organic acids also enhance plant tolerance to biotic and abiotic stressors through osmoregulation and potent antioxidant capacity^41^. Therefore, studying the organic acid content of spinach under abiotic stress to understand its nutritional value is crucial. In this study, nitrate stress increased oxalic, tartaric, and malic acid contents. Shams et al.^42^ showed that 100 mM NaCl increases oxalic and tartaric acid levels in lettuce plants. This finding is consistent with the results of this study. This may be because organic acids are involved in several underlying mechanisms, such as scavenging free radicals to protect cells from ROS-related damage, maintaining normal cellular activity. FA increased tartaric, malic, and citric acid contents under nitrate stress, providing sufficient substrates for the TCA cycle and amino acid biosynthesis and ensuring the normal production of energy and amino acids^26^. In addition, nitrate stress reduced the ascorbic acid content in this study, which is the same finding observed in wheat by Billah et al.^43^. FA application significantly increased the ascorbic acid content. This might be because FA has antioxidant properties that activate the plant antioxidant system to reduce the oxidative depletion of ascorbic acid and promote its regeneration. Similarly, FA might regulate gene expression to increase the transcription level of genes related to ascorbic acid synthesis, increasing its content. Thus, FA alleviates the inhibition of nitrate stress to some extent and promotes nutrient accumulation in spinach.

Mineral nutrients are essential for plant growth and development and play various physiological functions in plants, such as participating in the composition of the cell structure, regulating enzyme activity, maintaining electrochemical balance, influencing photosynthesis and energy metabolism, and synthesizing hormones^44^. Among them, cuprum, zinc, and other mineral elements are vital components of many enzymes in the human body that can promote metabolic processes and maintain normal physiological functions of the human body. Ferrum is an essential component of hemoglobin that promotes the transportation and utilization of oxygen and maintains normal respiration and energy metabolism in the human body. Mineral elements, such as sodium, potassium, and magnesium, maintain nerve and muscle functions and keep the body moving and feeling properly. Additionally, biotic and abiotic stressors can affect the pathways of mineral element uptake by the plant root system, affecting plant growth, development, and nutrient accumulation. In this study, nitrate stress increased N, K, and Ca contents in spinach, which is consistent with the results of Zhang et al.^45^ in cucumber. This might have been caused by the application of excess potassium and calcium nitrates in the nutrient solution; however, FA application inhibited the enhancement of their content. Zhang et al.^45^ showed that nitrate stress reduces phosphorus and magnesium levels. This is consistent with the findings of this study. The results showed that nitrate stress reduced the phosphorus, magnesium, natrium, ferrum, and zinc contents. This may be caused by various factors; for example, root cells are subjected to osmotic stress, ionic toxicity, and oxidative stress hazards in high salt concentration environments, which can cause a deficiency of mineral elements in the crop. Moreover, excessive accumulation of oxalic acid in plants can also affect their mineral nutrient uptake^46^, causing severe nutrient stress, which is consistent with the results of a previous study. However, FA application usually has the opposite effect, with only a further decrease in magnesium concentration and a significant increase in the contents of other elements. This indicates that FA can regulate the mineral element composition under nitrate stress. FA possibly enhances mineral element uptake by improving the environment of the plant root system; similarly, it might regulate the expression of genes related to the transport of mineral elements and optimize the distribution of mineral elements in plants^47^. Therefore, FA regulates the composition of mineral elements under adverse conditions, which is essential for plant growth and for improving the yield and quality of crops. A limitation of this study might be that the environmental conditions are relatively singular, which is different from the actual complex environment in the field and can only provide a theoretical basis for actual production and planting. Thus, the results of this study should be applied to field trials in future studies to explore the challenges in actual production, optimize the experimental operation and planting mode, and conduct a more in-depth investigation of the molecular mechanisms and signal transduction pathways to provide a theoretical basis for regulation.

## Conclusion

This study provides insights into the reactions of metabolic products and changes in the mineral element content of spinach upon FA application under nitrate stress. We found that FA promoted the accumulation of nutrients such as amino acids, phenolic acids, organic acids, and mineral elements under nitrate stress, reducing the adverse effects of stress on spinach plants. Therefore, growers can improve the nutritional quality and value of spinach through FA application under nitrate stress conditions. This study supports a new strategy for effectively mitigating adverse stressors and improving the nutritional quality of crops under nitrate stress.

## Materials and methods

### Plant materials and experimental design

The spinach variety used was Fire Phoenix 119 (Tuochetou International Co., Ltd., Hebei, China). We selected evenly-sized spinach seeds for the germination experiments. Germinated seeds were planted in porous trays containing a 3:1 ratio of vermiculite to perlite as seedling substrate. Subsequently, they were cultured in an artificial climate chamber (relative humidity was 70–80%; day and night temperature were 20 °C and 16 °C; light 12 h [20,000 Lx], dark 12 h). When the seedlings had four leaves and one heart, they were transplanted into a hydroponic box containing a Hoagland nutrient solution.

**Fig. 9:**
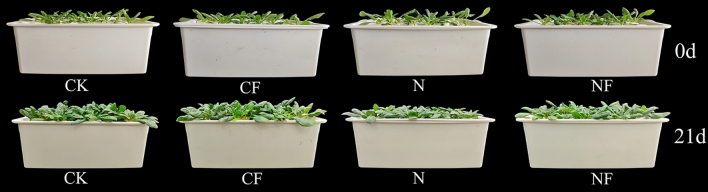
Photographs of the material before the start of the experimental treatments and before harvest. Control (CK), 0.15% FA (CF), 150 mM NO_3_^−^ (N), 0.15% FA + 150 mM NO_3_^−^ (NF).

Uniformly sized spinach plants were selected 20 days after transplantation for the treatment. The following four treatments were used: Normal control (CK), 0.15% FA (CF), 150 mM NO_3_^−^ (N), and 0.15% FA + 150 mM NO_3_^−^ (NF). The NO_3_^−^ concentration in the CK was 15 mM, and excess NO_3_^−^ in the stress treatment was provided using Ca (NO_3_)_2_·4H_2_O (37.5 mM) and KNO_3_ (75 mM), half each. The pH was maintained at approximately 6.0. Based on the normal nitrate ion concentration, the required amount of nitrate ions was added to achieve the desired treatment concentration. At the beginning of the nitrate treatment, 1/3 of the total remaining NO_3_^−^ concentration was added daily for 3 days until the set concentration was reached to prevent salt stimulation. The nutrient solution (5 mM KNO_3_, 5 mM Ca (NO_3_)_2_·4H_2_O, 2 mM MgSO_4_·7H_2_O, 1 mM KH_2_PO_4_, 0.045 mM H_3_BO_3_, 0.01 mM MnCl_4_·4H_2_O, 0.8 μM ZnSO_4_·7H_2_O, 0.4 μM Na_2_MoO_4_·2H_2_O, 0.3 μM CuSO_4_·5H_2_O, and 0.02 mM EDTA·Na-Fe) was replaced every 7 days. After 2 days of stress treatment, FA was sprayed uniformly on the leaf surface and back, and the control was sprayed with equal amounts of water. They were sprayed thrice (once every 7 days), sampled 4 days after the last spraying (total duration of 21 days; Fig. 9), and the relevant indices were measured.

### Determination of amino acids

Spinach sample preparation and LC–MS analysis of amino acids were performed following the method described by Jin et al.^48^ with slight modifications. We used 20 amino acid monomer standards that were obtained from Merck and Sigma (Sigma-Aldrich GmbH, Sternheim, Germany). Quantitative analysis was conducted using the external standard method, and the standard curve was generated using Agilent MassHunter workstation software. Fresh spinach leaf samples (15 g) were chopped and placed in liquid nitrogen for rapid freezing; subsequently, they were placed in an ultra-low temperature refrigerator at −80 °C for 1 h, placed in a freeze-dryer (LyoQuest-85, Telstar Technologies, Barcelona, Spain) for 72 h, and removed to be ground into a powder using a grinder (SD-YM1502, WEILI, Langfang, China). Lyophilized spinach powder (0.1 g) was weighed into a 2-mL centrifuge tube, and hydrochloric acid solution (2 mL, 0.5 M) was added for extraction. The samples were mixed using a vortex mixer (MX-S, Scilogex, San Diego, California, USA) at 8,000 rpm for 20 min and extracted using ultrasound (SB-800 DT, NingBo Scientz Biotechnology Co., Ltd., Ningbo, China) for 20 min at room temperature. After sonication, the samples were centrifuged using a centrifuge (TGL-18 M; Shanghai Lu Xiangyi Centrifuge Instrument Co., Ltd., Shanghai, China) at 20,000*g* for 20 min at room temperature. Finally, the supernatant was passed through a 0.22-μm water phase membrane filter, and the sample (5 μL) was injected into HPLC–MS (LC–MS, Agilent 1290–6460, CA, USA) for quantitative analysis.

HPLC conditions are as follows: the chromatographic column was Agilent InfinityLab Poroshell 120 HILIC-Z (2.1 × 100 mm, 2.7 μm); the column temperature was set to 25 °C; mobile phase A comprised water and ammonium formate stock solution, and the ratio was 9:1; mobile phase B comprised acetonitrile and ammonium formate stock solution, and the ratio was 9:1 (200 mM ammonium formate stock solution was prepared with water, pH = 3, and the final concentrations of mobile phases A and B were 20 mM); flow rate was 0.5 mL min^−1^; the total run time was 15 min. The MS source conditions are as follows: the capillary voltage was 1,500 V; the sheath gas flow rate was 12.0 L min^−1^; the sheath gas temperature was 390 °C; the temperature of the dryer was 330 °C; the atomizer was 35 psi; the gas flow rate was 13.0 L min^−1^; the ionization mode was ESI positive ion mode.

### Determination of phenolic acids

The phenolic acid fractions in spinach leaves were analyzed using HPLC. The lyophilized spinach powder (0.1 g) was placed in a 5-mL centrifuge tube, methanol (2 mL) was added, and the mixture was left at room temperature for 1 h for extraction. The samples were centrifuged at 4 °C and 6,600*g* for 10 min, and the supernatant was collected, passed through a 0.22-μm organic phase filter membrane, and left to be measured. The samples were analyzed with HPLC using a symmetric C18 column (250 × 4.6 mm, 5 μm; Waters Corp., Milford, MA, United States). The chromatographic conditions are as follows: flow rate was 1.1 mL min^−1^; injection volume was 10 μL; mobile phase A was methanol and mobile phase B was 1% acetic acid (v/v); the column temperature was 30 °C. Gradient elution was performed. The compounds were detected at 240, 280, and 322 nm. The compounds were identified based on the retention times of the standards and were quantitatively analyzed according to standard curves (the standards were purchased from Shanghai Yuanye Biotechnology Co., Ltd., Shanghai, China).

### Determination of organic acids

A fresh spinach sample (0.2 g) was placed in a mortar, and ultrapure water (5 mL) was added to grind it into a homogenate. It was decanted into a 10-mL centrifuge tube; the mortar was rinsed twice with ultrapure water (2.5 mL). The rinse solution was transferred to a centrifuge tube and centrifuged for 10 min at 4 °C and 8,500*g*. Subsequently, the supernatant was passed through a 0.22-μm aqueous filtration membrane and used in determining organic acid components using HPLC. The detecting instrument was a high-performance liquid chromatograph equipped with an ultraviolet detector (Agilent 1260 Infinity II, Agilent Technologies, USA). It was analyzed on a chromatographic column X-Peonyx AQ-C18 (250 × 4.6 mm; FeiniGen instrument, China). The detection wavelength was 210 nm; the injection volume was 10 μL; the column temperature was 30 °C; the mobile phase was sodium dihydrogen phosphate (0.2 mM); isocratic elution was performed; the flow rate was 1.2 mL min^−1^.

### Determination of mineral elements

To determine the mineral element content in the aboveground parts of spinach, fresh spinach samples were placed in an oven at 105 °C for 30 min to kill the green and dried at 80 °C until a constant weight was achieved. The dried sample was removed, ground into powder, and placed in self-sealing bags for spare use. The dried spinach sample (0.2 g) was placed in a 150-mL triangular flask, and concentrated sulfuric acid (5 mL) was added. The mixture was shaken gently and left at room temperature overnight. The sample was digested through wet digestion using H_2_SO_4_-H_2_O_2_ at 400 °C on a hot plate until the digestive fluid was colorless or clear, and the triangular flask was removed and cooled to room temperature. The digestive fluid was diluted with ultrapure water and filtered into a 100-mL volumetric flask, and the filtrate was collected to measure the mineral elements. Elemental nitrogen in the samples was determined using an Automatic Kjeldahl Analyzer (K1100; Haineng Future Technology Group Co., Ltd., Jinan, China). The phosphorus content was determined using the molybdenum antimony colorimetric method. The potassium, sodium, calcium, magnesium, manganese, ferrum, copper, and zinc levels were determined using a ZEEnit 700P atomic absorption spectrometer (Analytik Jena AG, Germany).

### Statistical analysis

Three replicates were performed for each assay, and the results were reported as mean and standard error. The SPSS software package was used to analyze the data. A one-way analysis of variance was performed to determine statistically significant differences between treatments, followed by Duncan’s multiple-range test (*p* < 0.05). Origin 2022 was used to draw bar charts with standard error bars and for correlation, principal component (PCA), and hierarchical clustering analysis (HCA).

## Data Availability

The datasets generated during and/or analysed during the current study are available from the corresponding author on reasonable request.
